# Cucurbit [7] uril encapsulated cisplatin overcomes resistance to cisplatin induced by Rab25 overexpression in an intraperitoneal ovarian cancer model

**DOI:** 10.1186/s13048-015-0189-4

**Published:** 2015-09-18

**Authors:** Natividad Gomez-Roman, Fiona McGregor, Nial J. Wheate, Jane A. Plumb

**Affiliations:** Institute of Cancer Sciences, Wolfson Wohl Translational Cancer Research Centre, University of Glasgow, Bearsden, Garscube Estate, Glasgow, G61 1QH, Scotland UK; Faculty of Pharmacy, The University of Sydney, Sydney, NSW 2006 Australia

## Abstract

**Background:**

Ovarian cancer is the most fatal of gynaecological malignancies, usually detected at a late stage with intraperitoneal dissemination. Appropriate preclinical models are needed that recapitulate both the histopathological and molecular features of human ovarian cancer for drug-efficacy analysis.

**Methods:**

Longitudinal studies comparing cisplatin performance either alone or in a novel cisplatin-based delivery-system, cucurbit[7]uril-encapsulated cisplatin (cisplatin@CB[7]) were performed on subcutaneous (s.c.) and intraperitoneal (i.p.) xenografts using the human ovarian cancer cell line A2780 stably expressing the small GTPase Rab25, which allows A2780 intraperitoneal growth; and luciferase, to allow tumour load measurement by non-invasive bioluminescent imaging.

**Results:**

Rab25 expression induced cisplatin resistance compared to the parental cell line as assessed by the MTT assay *in vitro*. These findings did not translate *in vivo*, where cisplatin resistance was determined by the microenvironment. Subcutaneous xenografts of either parental A2780 or cisplatin-resistant Rab25-expressing A2780 cells presented similar responses to cisplatin treatment. In contrast, increased cisplatin resistance was only detected in i.p. tumours. Treatment of the cisplatin-resistant i.p. model with the novel cisplatin@CB[7] delivery system resulted in a substantial reduction of i.p. tumour load and increased necrosis.

**Conclusions:**

Poor clinical performance of novel chemotherapeutics might reflect inappropriate preclinical models. Here we present an ovarian i.p. model that recapitulates the histopathological and chemoresistant features of the clinical disease. In addition, we demonstrate that the novel cisplatin-delivery system, cisplatin@CB[7] may have utility in the treatment of drug-resistant ovarian human cancers.

## Background

Ovarian cancer is the most lethal of all gynaecological cancers, mostly because it is detected at late clinical stages, when the treatment is less effective [1]. Late stage ovarian cancer presents with widespread peritoneal dissemination and ascites. Treatment usually involves aggressive cytoreductive surgery, and modern combination chemotherapy (platinum and paclitaxel) or i.p. cisplatin based-chemotherapy [2–6]. Lack of an adequate screening test for early disease detection and the rapid progression to chemoresistance have prevented appreciable improvement in the five year survival rate of patients with ovarian cancer [7]. Dissemination of single tumour cells into the peritoneal cavity is the major cause of tumour recurrence even after complete resection of the primary solid tumour [8, 9]. Currently, there is no effective treatment for peritoneal carcinomatosis [10]. Preclinical models that mimic the complexity of tumour behaviour and microenvironment in patients are essential for the evaluation of novel chemotherapeutics. These preclinical models include models of spontaneous ovarian carcinoma in experimental animals [11, 12] or genetically modified animals [13, 14]. However, the long latency to tumourigenesis and the heterogeneity in the timing of advanced tumour development makes the use of these models in preclinical studies challenging. Ovarian cancer cell lines derived from ascites or primary ovarian tumours have been used extensively and can be very effective for studying the processes controlling growth regulation and chemosensitivity [15]. However, most of these studies rely on subcutaneous xenografts, that do not provide the relevant tumour microenvironment which that might influence treatment response [9]. Tumour formation resulting from peritoneal implantation of human ovarian carcinoma cells holds promise as pre-clinical models of human ovarian cancer, as they are relatively rapid to generate and develop in the relevant microenvironment. Expression of Rab25, a member of the Ras superfamily of GTPases, in the ovarian cancer cell line A2780, allows these cells to invade and grow in the peritoneum of mice, resulting in tumour growth accumulation and death recapitulating the peritoneal disseminated disease in humans [16]. Rab25 was discovered to be the driving event of the 1q22 genomic amplification associated with poor disease-free survival rate following surgical and chemotherapy procedures [16]. Its enforced expression in ovarian and breast cancer cell lines induced cell number through reduced apoptosis after multiple stress conditions, including UV radiation and exposure to paclitaxel [16]. These results suggest that Rab25 expression might also influence cisplatin resistance.

In the present study, we have developed a model of peritoneal ovarian carcinomatosis in athymic immunodeficient mice using the A2780 cell line overexpressing Rab25 that can be monitored longitudinally through by bioluminescence. We show that Rab25 expression in this cell line induces cisplatin resistance *in vitro* and *in vivo*, although the *in vivo* resistance to treatment is also determined by the localisation of the tumour. In addition, we confirm increased anti-tumour activity of the novel cisplatin delivery system Cucurbit[7]uril (cisplatin@CB[7]) compared to free cisplatin in the i.p. model which exceeded that observed in the s.c. model. Cucurbit[n]uril is a barrel-shaped molecule, containing a hydrophobic cavity, formed by the acid catalysed condensation of glycoluril and formaldehyde. Cucurbit[n]urils can be synthesised in a variety of sizes (n=5, 6, 7, 8 and 10), and are capable of encapsulating smaller molecules within their cavities. Our results demonstrate that cisplatin@CB [7] may have utility in the treatment of drug-resistant ovarian human cancers and warrant further investigation.

## Results

### Development of a bioluminescent ovarian cancer model in athymic mice

**Fig. 1:**
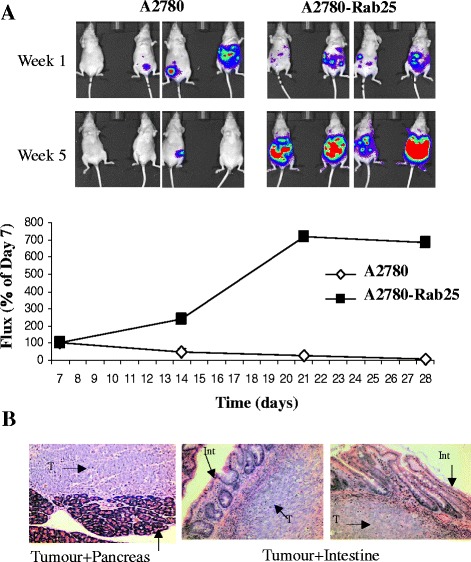
A2780 cells expressing Rab25 produce intraperitoneal disease in nude mice. **a** Nude mice were injected i.p. with 5 × 10^6^ of A2780 or A2780-Rab25 cells stably expressing luciferase and monitored over time by measuring luciferase expression after s.c. administration of luciferin (30 mg/ml) using an IVIS-50 imaging system. **b** H&E images of tumours taken from mice at 28 days after inoculation. T = tumour; Int = intestine

To generate an i.p. ovarian cancer model where tumour burden can be monitored and measured non-invasively, we developed an A2780-Rab25 stable cell line expressing the firefly luciferase gene under the control of the ubiquitin C promoter using a lentiviral gene delivery system. Luciferase-expressing cells injected into the peritoneal cavity of mice allow their imaging in the living animal after luciferin injection using the Xenogen IVIS50 system. Intraperitoneal injection of A2780 cells stably expressing Rab25 and luciferase resulted in tumour growth accumulation and death of all the animals injected, whereas only 20 % of the animals injected with the parental cell line expressing empty vector produced disease (Fig. 1a). The growth of these tumours was followed in longitudinal studies where bioluminescence quantification of the tumour burden was performed in real time in a single animal (Fig. 1a). The tumours localised to the peritoneum, presenting one or two big tumours invading the intestine, liver, pancreas and/or stomach (Fig 1b), and several small tumours spread throughout the peritoneal cavity.

### Rab25 expression increases cisplatin resistance *in vitro*

**Fig. 2:**
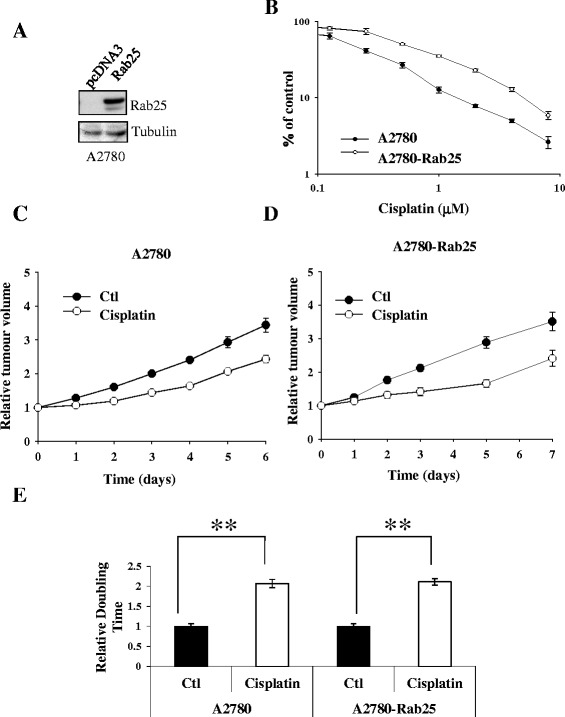
Rab25 expression increases cisplatin resistance of the human ovarian cancer cell line A2780. **a** Cell extracts were analysed by Western Blot to verify the expression of Rab25 after selection with geneticin. Tubulin expression was used as loading control. **b** The *in vitro* cytotoxicity of cisplatin was compared in the empty-vector expressing A2780 and Rab25-expressing A2780 by MTT assay. IC_50_ is defined as the concentration of drug required to inhibition of cell growth by 50 %. **c** and **d** The *in vivo* cytotoxicity of cisplatin was analysed for seven days by caliper measurements in either A2780 (**c**) or A2780-Rab25 (**d**) s.c. xenografts following a single dose of cisplatin at its maximal tolerated dose (6 mg/Kg). **e** Graphical representation of relative doubling time of A2780 and A2780-Rab25 s.c. xenografts depicting time required for cisplatin-treated xenografts to reach same size as control. p<0.05, calculated by *t*-test

The A2780 cell line presents high sensitivity towards cisplatin (IC_50_ 0.1 μM by MTT). Rab25 was discovered in tumours that did not respond to chemotherapy, implicating Rab25 as a potential oncogene regulating chemoresistance. Expression of Rab25 in the A2780 cell line increased a 2.8 fold cisplatin resistance compared to vector-only cells as measured by MTT assay (A2780-Rab25Luc IC_50_ = 0.555 ± 0.058 μM to A-Luc IC_50_ = 0.1973 ± 0.0217 μM, Fig. 2b). To investigate if Rab25 expression could affect the cisplatin sensitivity *in vivo*, s.c. xenografts of either the ovarian parental cell line A2780 or the A2780-Rab25 cell line were compared. Once tumours were established, the mice were treated with a single dose at the maximum tolerated dose (MTD) for cisplatin of 6 mg/kg and tumour growth was monitored using caliper measurements. The Rab25-expressing and the parental A2780 s.c. xenografts presented a very similar response to cisplatin, demonstrating that the Rab25 conferred chemoresistance to cisplatin was lost *in vivo* (Fig. 2c).

### Evaluation of the i.p. model for cisplatin treatment

**Fig. 3:**
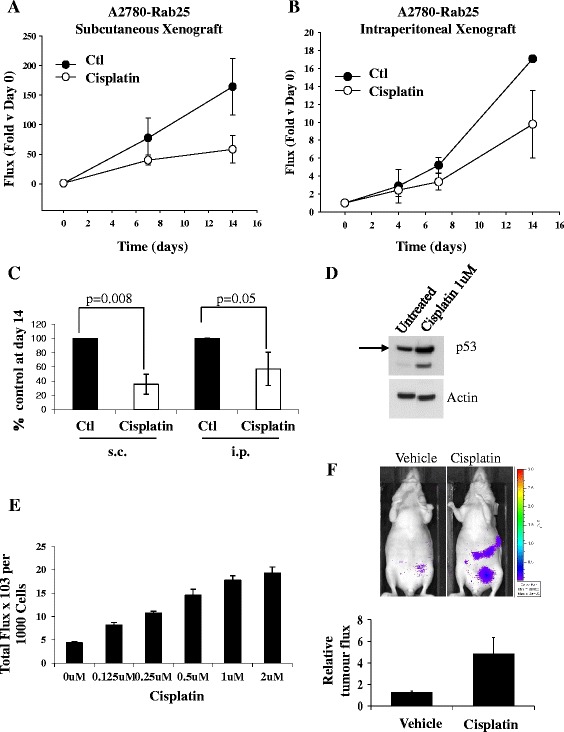
Comparison between cisplatin treatment in s.c. and i.p. xenografts. **a** A2780-Rab25 cells were injected at 2 × 10^6^ subcutaneously (s.c.) or (**b**) 5 × 10^6^ intraperitoneally (i.p.) in nude mice. Mice were left for approx. 1 weeks (s.c.) or 3–4 weeks (i.p.) to allow tumours to develop. Cisplatin (6 mg/kg, Cisplatin) was injected s.c. on day 1. Luciferase signal was monitored over time beginning day 0. SEM bars are shown. **c** Luminescent signal (arbitrary units) at day 14 compared to their relative luminescent starting signal at day zero. **d** *In vitro* increase in endogenous p53 expression following treatment of cells with 1uM cisplatin. **e** Overnight culture of cells on 24 well plates was incubated for 24 hours with cisplatin (4 wells per concentration). Luciferase activity was measured using the Xenogen IVIS and the cells counted. Luciferase activity is expressed as total flux × 10^3^ per 1000 cells. **f** Mice (12) were injected i.p. with 5 × 10^6^ A2780Rab25p53-Luc cells. Tumours developed over 27 days. The mice were imaged before and 24 hours after treatment with cisplatin 6 mg/Kg i.p. (6 mice in each group). Representative example of bioluminescent imaging signal. Graph depicts relative increase in p53 response luminescent signal

To investigate whether tumour microenvironment determines response to cisplatin in ovarian cancer xenografts, A2780-Rab25 cells were injected into nude mice either subcutaneously or intraperitoneally. Establishment of the tumours was very different in each model. One week after subcutaneous injection of the cells, the tumours were ready to be monitored by bioluminescence and the mice were treated at this time with cisplatin at the MTD and imaged three times during the course of the experiment (Fig. 3a). Slower development of tumours was observed in the i.p. model. After i.p. injection of cells, the mice were imaged once weekly until the appearance of bioluminescent signal was detected, and the signal was above 8x10^5^ flux (around three to four weeks; data not shown). At this time, the animals were treated with cisplatin at the MTD, and imaged three times for the same period as s.c. xenografts (14 days; Fig. 3b). Both s.c. and i.p. tumours presented growth delays after cisplatin treatment. While the rapid growing s.c. xenografts reached only 35 % of the signal to that of control at day 14, the slow growing i.p. xenografts reached ~60 % at the same time (Fig. 3). These results demonstrate that the s.c. xenografts are more sensitive to cisplatin than the i.p. tumours.

### Induction of p53 by cisplatin in the A2780-Rab25 i.p. xenografts

The increased chemoresistance observed in the i.p. xenografts could be explained by reduced drug penetrance to the i.p. tumours. To assess if cisplatin was reaching the i.p. xenografts, we developed an A2780-Rab25 cell line stably expressing a luminescent p53-luciferase reporter, containing the luciferase gene under the control of a p53 regulated promoter. Cisplatin produces DNA adducts [17] which stabilise and activate p53, inducing the expression of its downstream targets genes involved in apoptosis (Zamble, 1998). The A2780-Rab25 cell line expresses normal p53 that responds to cisplatin treatment (Fig. 3d), as represented by an increase in full-length p53 as well as an increase in a p53 splice variant (lower band). Exposure to different concentrations of cisplatin for 24 hrs induced a dose–response activity of the p53-driven luciferase reporter *in vitro* (Fig. 3e). To analyse if we could monitor p53 activation *in vivo* after cisplatin treatment in the i.p. model, we generated i.p. xenografts with A2780-Rab25 stably expressing the p53-luciferase reporter. Four weeks after cell injection, mice were treated with cisplatin at the MTD. An increase in luminescence was observed in the tumours compared to control (Fig. 3f), confirming that cisplatin is indeed reaching the i.p. tumours.

### Activity of Cisplatin@CB[7] on the A2780-Rab25 i.p. model

The delivery of platinum drugs can be improved through their encapsulation, by taking advantage of the enhanced permeability and retention effect and/or protection from degradation and deactivation. Cucurbit[7]uril (CB[7]), is a rigid macrocycle made from the condensation of glycoluril and formaldehyde [18, 19] and is capable of storing and releasing small molecules for controlled drug release. Encapsulation within CB[7] also provides steric hindrance to degradation by thiol amino acids, peptides and proteins for both multinuclear and mononuclear platinum drugs [20].

The effect of CB[7] encapsulation on the cytototoxicity of cisplatin was evaluated *in vitro* in the human ovarian A2780 cell line and the cisplatin-resistant A2780-Rab25 cell line. Determination of the *in vitro* cytotoxicity was performed by MTT assays. No significant difference in cytotoxicity between free cisplatin and cisplatin@CB[7] was observed in either cell line (Table 1). These results are encouraging as encapsulation of cisplatin can lead to large or complete loss of *in vitro* cytotoxicity. Recently, we have shown that cisplatin@CB[7] is just as effective on A2780 s.c. tumours compared with free cisplatin, and in the cisplatin-resistant A2780/cp70 tumours CB[7]-encapsulated cisplatin markedly slows tumour growth. The ability of cisplatin@CB[7] to overcome resistance *in vivo* appears to be a pharmacokinetic effect [21].

**Table 1:** Comparison between cisplatin and cisplatin@CB[7] toxicty by MTT assay. The *in vitro* cytotoxicity of cisplatin and cisplatin@CB[7] in the human ovarian cancer cell line A2780 and its cisplatin-resistant cell line A2780-Rab25. IC_50_ is defined as the concentration of drug required to inhibition of cell growth by 50 % ovarian cancer cell line A2780 and its cisplatin-resistant cell line A2780-Rab25. IC_50_ is defined as the concentration of drug required to inhibition of cell growth by 50 %

Cell line	IC_50_ (μM) Cisplatin	Cisplatin@CB[7]
A2780	0.16 ± 0.01	0.16 ± 0.001
A2780-Rab25	0.47 ± 0.04	0.42 ± 0.02

**Fig. 4:**
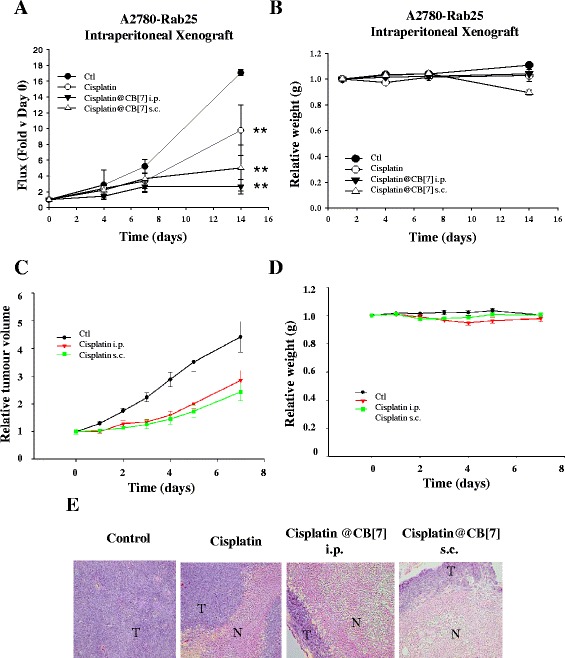
Cisplatin@CB[7] retains its enhanced activity in the disseminated ovarian cancer model. **a** Delay on intraperitoneal tumour growth over 14 days of A2780-Rab25 i.p. xenografts following injection on day 1 of saline (Ctl), cisplatin at 6 mg/kg, cisplatin@CB[7] at 34 mg/kg (equivalent to 6 mg/kg of cisplatin), and subcubtaneous injection of cisplatin@CB[7] at 34 mg/kg (equivalent to 6 mg/kg of cisplatin). SEM bars are shown. **b** Relative body weights over fourteen days after treatment. **c** Comparison between cisplatin treatment injected through s.c. or i.p. route in A2780 subcutaneous xenografts measured for tumour volume by calipers after cisplatin injection on day 0. Body weight was monitored for the duration of the experiment. SEM bars are shown. **d** Relative body weights over fourteen days after treatment. **e** Representatitve H&E images of orthotopic ovarian tumours (T) from the control, cisplatin, cisplatin@CB[7] injected i.p. and cisplatin@CB[7] injected s.c. cohorts. Necrotic regions (N) are observed in all cisplatin cohorts

We then analysed the *in vivo* cytotoxicity of cisplatin@CB[7] using the A2780-Rab25 i.p. xenografts by bioluminescence. Once the tumours could be detected, the mice were treated on day 1 with a single injection of the MTD of cisplatin (6 mg/kg) or cisplatin@CB[7] at an equivalent cisplatin dose (34 mg/kg which equates to a cisplatin dose of 6 mg/kg). This dose was well tolerated by the mice, as indicated by their stable body weights over fourteen days (Fig. 4b). The mice were monitored by bioluminescence on day zero, three, six and thirteen after treatment injections. Tumours in the control group increased in luminescent signal by 17.1 fold compared to their relative luminescent starting signal at day zero (Fig. 4a). Conversely, both free cisplatin and cisplatin@CB[7] displayed a significant ability to retard tumour growth (Fig. 4a). As previously shown, the signal of the cisplatin treated tumours was 48.8 ± 21 % less than that of the vehicle cohort (Ctl) at the last time point. cisplatin@CB[7] tumour growth retardation was significantly more pronounced than free cisplatin; presenting an 84.4 ± 5 % reduction in signal to that of control mice at the last time point. To verify that the effect observed by cisplatin@CB[7] on the growth of tumours was independent to the route of administration, a fourth group of mice bearing i.p. A2780-Rab25 tumours was injected subcutaneously with cisplatin@CB[7]. The signal of the s.c. cisplatin@CB[7] treated mice was slightly higher than that of i.p. cisplatin@CB[7], with a signal of 29.3 ± 16 % to that of the control mice at day 14. This result correlates with our previous validation for cisplatin treatment where injection via i.p. or s.c. route gives a very similar response to tumour growth retardation compared to the control mice (Fig. 4c). No changes in relative body weight all cohorts (vehicle or cisplatin/cisplatin@CB[7] cohorts) demonstrated that this novel compound is well tolerated by mice (Fig. 4b).

### Pathology of the disseminated disease following cisplatin and CB[7]-cisplatin treatment

The disease observed in the control group had different characteristics compared to that of the treatments groups. The control group was characterised by disseminated disease with widespread small tumours growing in the peritoneum and the occasional appearance of one or two medium size tumours attached to organs (6 out of 6 mice, Table 2). Conversely, both free cisplatin and cisplatin@CB[7] presented little disseminated disease. Instead, the presence of one or two very big tumours attached to organs was observed. The tumours were extracted, formalin-fixed, paraffin-embedded and analyzed by hematoxylin and eosin stain. Examination of the tumours showed extensive areas of necrosis in the tumours from the treated mice, presenting larger necrotic areas in the cisplatin@CB[7] group compared to the free cisplatin group (Table 2 and Fig. 4e), confirming its enhanced activity.

**Table 2:** Peritoneal Disease observed after cisplatin or cisplatin@CB[7] treatment. Pathological observations of the i.p. tumours extracted from the control, cisplatin and cisplatin@CB[7] groups. Size of tumour was determined as follows: + < 0.5 cm; ++ >0.5 cm < 1 cm; +++ >1 cm in diameter. Dissem. D. = Disseminated disease was determined as >3 small tumours spreaded throughout the peritoneal cavity

Treatment	Dissem. Disease	Attached to organ	Size of Tumours	Tumour for H&E	% of Necrotic Area
Control	6/6	4/6	+	3/6	11.66 ± 8.924 %
Cisplatin i.p.	3/6	3/6	++	4/6	32 ± 6.75 %
Cisplatin@CB[7) i.p.	2/6	4/6	+++	6/6	47.85 ± 10.11 %
Cisplatin@CB[7) s.c.	0/5	5/5	+++	4/5	47.5 ± 18.35 %

## Discussion

Ovarian cancer commonly presents at a late stage with extensive metastatic deposits in the peritoneum and abdominopelvic organs. In order to properly evaluate novel therapies and delivery systems for this type of disease, preclinical models that recapitulate better the human presentation are essential. Rab25 expression in ovarian cancer cell lines results in a more invasive phenotype *in vitro* and *in vivo*, with extensive metastatic deposits in the peritoneum and abdominopelvic organs [16] and Fig. 2). Here, we show a modest increased cisplatin resistance induced by Rab25 expression in the ovarian cancer cell line A2780 *in vitro*. This modest increase parallels observations in the clinic, where resistance can be gained by just a small order of magnitude. Induction of cisplatin-resistance via overexpression of Rab25 in the ovarian cancer cell line SKOV-3 has also been recently reported. Our results expand our understanding of cisplatin resistance, where resistance to treatment also depends on the microenvironment, as demonstrated by the increased cisplatin resistance of the i.p. tumours compared to no changes in sensitivity in a subcutaneous model. For the past two decades, s.c. xenografts have constituted the major pre-clinical screen for the development of novel cancer therapeutics. Despite limitations, these models have identified clinically efficacious agents, and remain the ‘workhorse’ of the pharmaceutical industry. However, they have received criticism as good animal models for pre-clinical drug evaluation as they lack the proper microenvironment which might determine therapeutic response [22]. Our results clearly demonstrate the role that the microenvironment plays on therapeutic response, exemplifying further why we require models that recapitulate the human presentation.

Another disadvantage of the s.c. models are the way that these tumours are monitored. Caliper measurements are error-prone due to variability in tumour shape, skin thickness and subcutaneous fat layer thickness. Observer subjectivity and differences in the compressibility of the tumour can also easily lead to increased errors [23]. In addition, some subcutaneous xenografts are known to develop necrotic centres, giving an underestimated value of drug effect [24]. With the advent of non-invasive visible light imaging technology, biological processes such as tumour load can be monitored in live animals through time, without restriction in their localisation. Because of its sensitivity, luminescence also allows to initiate drug evaluation much earlier than with calipers, which permits analysis of tumour growth for longer periods of time. Our data illustrates that bioluminescence quantification is a reliable method to monitor tumour burden allowing for direct comparison between a disseminated model and the s.c. model and demonstrates that the microenvironment determines tumour therapeutic response. Furthermore, using this system we have been able to identify the therapeutic benefit of a novel cisplatin drug delivery system. These results suggest that the establishment and growth of s.c. xenografts differs from that of xenografts in the peritoneal cavity. Our results might explain the poor clinical performance observed of novel cancer drugs in the clinic which have been previously evaluated in unsuitable pre-clinical models.

## Conclusions

Continued advancement in cancer research depends on the use of adequate experimental animal models in order to develop appropriate therapies that will translate better into the clinic. Our data indicate that the bioluminescent i.p. model resembles the clinical outset and might improve pre-clinical screening. We also confirm increased anti-tumour activity of the novel cisplatin@CB[7] compared to free cisplatin in the i.p. model. Our results demonstrate that cisplatin@CB[7] may have utility in the treatment of drug-resistant ovarian human cancers and warrant further investigation.

## Methods

### Materials

Cisplatin, MTT [3-(4,5-dimethylthiazol-2-yl)-2,5-diphenyltetrazolium bromide] were obtained from Sigma-Aldrich Co. Ltd (Dorset, UK). Luciferin was obtained from Caliper Lifesciences Ltd (Wellfield, UK). G418S Sulphate solution was obtained from ForMedium Ltd (UK). Blasticidin, Lipofectamine 2000, Virapower Promoterless Lentiviral Gateway Kit were obtained from Invitrogen Co. (Paisley, UK).

### Preparation of cisplatin@CB[7]

Cisplatin (Sigma-Aldrich) and CB[7] were stirred together in hot water until dissolved, then stirred for a further 3 h before being either freeze dried or rotary evaporated to dryness. The water content of the cisplatin@CB[7] complex was then determined by elemental analysis and found to be between 5 and 13 water molecules per batch. These waters of crystallisation were taken into account when calculating the molecular mass of cisplatin@CB[7] and the subsequent concentrations of each batch in solution before administration.

### Luciferase reporters

Two luciferase lentiviral constructs were produced (pLenti-UbCp-Luc and pLenti-p53 RE-Luc) using the ViraPower Promoterless Lentiviral Gateway Expression System according to manufacturers’ instructions for the generation of stable cell lines expressing luciferase under the control of the Ubiquitin C promoter or a p53 reporter containing p53 binding regions. For the Ubiquitin C-Luciferase lentiviral construct, the pENTR™5’-UbCp plasmid containing the Ubiquitin C promoter (supplied in the kit) and the pENTR™/D-TOPO®-Luciferase plasmids were combined with the promoterless pLenti6/R4R2/V5-DEST destination vector (Invitrogen, cat. No. K5910-00) into which the promoter and gene of interest were transferred using MultiSite Gateway® Technology (no ligase, post-PCR procedures or restriction enzymes required). The luciferase gene was amplified from plasmid pGL4-CMVLuc (Promega) using the following primers: Forward-5′ CACCATGGAAGACGCCAAA; Reverse-5′ AAACACGGCGATCTTTCCG and cloned into the pENTR™ Directional TOPO® plasmid (Invitrogen, cat. No. K2400-20). For the p53-Luciferase lentiviral construct, the p53 binding region was amplified from the p53 Luciferase Reporter Vector (Panomics, cat. No LR0057) using the following primers: Forward-5′ GGTACCGAGCTCTTA; Reverse-5′ GCT TTACCAACAC. The PCR product was cloned into pENTR 5′-TOPO TA plasmid (Invitrogen, cat. No. K591-10). The pENTR™5′TOPO®TA plasmid containing the p53 binding region (pENTR™5′TOPO®TA-p53RE) and the pENTR™/D-TOPO®-Luciferase plasmid were combined with the promoterless pLenti6/R4R2/V5-DEST destination vector (Invitrogen, cat. No. K5910-00) into which the promoter and gene of interest were transferred using MultiSite Gateway® Technology (no ligase, post-PCR procedures or restriction enzymes required) and lentiviral construct was produced through recombination. All PCR reactions were carried out with Proofstart DNA Polymerase (Qiagen) following manufacturer’s protocols. Cloning of the luciferase product into pENTR^™^-gene and of the p53 reporter into pENTR^™^-TOPO®TA was done according to manufacturers’ instruction (Virapower^™^ Promoterless Lentiviral Gateway® Kits). Cloning product was confirmed by sequencing with ABI PRISM 3130XL Genetic Analyzer (Applied Biosystems, Foster City, CA). For lentivirus production, transfection 3 μg of either pLenti-UbiquitinCpromoter-Luciferase and pLenti-p53 reporter-Luciferase constructs plus ViraPower™ Packaging Mix was performed following manufacturer’s instructions (Invitrogen cat. No. K5910-00). Viruses (lentivirus-p53RE-Luc or lentivirus-UbCp-Luc) were harvested 72 hr post transfection, filtered through 0.45 μm filter (Millipore, Bedford, MA) and re-suspended in fresh medium or array buffer. Titers of lentiviral preparations were determined using A2780 cells and were around 10^6^ IFU/ml. To infect cells using lentiviral vectors, cells were placed in a 6-well plate at a density of 5x10^7^ million cells/well the day before infection. The next day medium was removed and 2 ml lentivirus in cell culture medium was added in the presence of 2 μg/ml polybrene to initiate infection. Viruses were removed 24 hr after infection and fresh cell culture medium was added. After 24 hr, blasticidin (2.5 μg/ml) was added and selection performed for cells expressing luciferase.

### Cell lines

The human ovarian carcinoma cell line A2780 was obtained from Dr. R. F. Ozols (Fox Chase Cancer Centre, Pennsylvania, U.S.A.). Cells were maintained in Roswell Park Memorial Institute 1640 medium (Invitrogen) containing glutamine (2 mM) and foetal calf serum (10 %). The stable cell lines expressing Rab25 or empty vector were generated by selection of transfected cells with pcDNA3-Rab25 or pcDNA3 plasmid using Lipofectamine 2000 according to manufacturers’ instructions. Once stables cells were selected and analysed for Rab25 expression, they were transduced with a firefly luciferase reporter previously generated and clones were selected with blasticidin. Stable cell lines expressing Rab25 and luciferase were grown in the presence of geneticin (0.5 mg/ml) and blasticidin (2.5 μg/ml ). Cells were grown in the absence of drugs for 24 hours before in vivo experiments and for the duration of experiment for in vitro assays.

### Cytotoxicity assay

Drug sensitivity was determined by tetrazolium based chemosensitivity assay as described previously [25]. Briefly, cells were plated out at a density of 1x10^3^ per well in 96-well flat bottomed plates (IWAKI) and allowed to attach and grow for 2–3 days. They were exposed to cisplatin for 24 hr and fresh media was added on the following day. On the fourth day after treatment, MTT (50 μL, 5 mg/mL) was added to each well. Plates were incubated in the dark at 37 °C for 4 hr, media and MTT removed and the MTT-formazan crystals dissolved in dimethyl sulphoxide (200 μL/well). Glycine buffer (25 μL/well, 0.1 M, pH 10.5) was added and the absorbance measured at 570 nm in a multi-well plate reader (Model *Emax*, Molecular Devices Ltd., Wokingham UK).

Results are expressed in terms of the drug concentration required to kill 50 % of the cells (IC_50_) estimated as the absorbance value equal to 50 % of that of the control untreated wells.

### Tumour growth delay *in vivo*

Cell lines A2780 and A2780-Rab25 were established as xenografts in six to eight-week-old athymic female nude (MF1 nu/nu) mice. Monolayer cultures were harvested with trypsin/EDTA and resuspended in PBS. Animals were housed in autoclaved microisolator cages in an air-filtered laminar flow cabinet and were given food and water ad libitum. All procedures were performed under sterile conditions in a laminar flow hood.

### Ethical approval

Animal experiments were in compliance with all regulatory guidelines, as described in the Animals Act 1986 Scientific Procedures on living animals regulated by the Home Office in the United Kingdom.

For xenografts studies, 2x10^6^ cells were injected into the right flank of mice for the s.c. model and 5 × 10^6^ cells were injected in the peritoneal cavity for the i.p. model [16]. Two weeks later, tumour bearing mice were randomized into groups of six and tumour volume and body weight recorded daily for duration of experiment. Mice were injected i.p. with the maximum tolerated dose of cisplatin (6 mg/Kg) on day 1. Tumour volumes were estimated by caliper measurements assuming spherical geometry (volume = π/6x*d*^3^, where *d* is the mean diameter). For luminescence recording, mice were injected s.c. with 200 μl of the D-luciferin substrate (30 mg/ml). Following injection, mice were anaesthetised with isoflurane (3 % and 1 liter/min oxygen) and placed in the imaging system. Luminescence was recorded 10 min after luciferin injection with an exposure time of 1 min using the Xenogen IVIS50 imaging system from Caliper Life Sciences Ltd. (Wellfield, UK). Imaging was repeated weekly to allow estimation of tumour burden over time.

For i.p. xenografts, about 5x10^6^ cells were injected into the peritoneal cavity of mice and mice were imaged on a weekly basis.

Analysis of the bioluminescent images was performed by defining a set region of interest to be used in all animals. For the disseminated model, the signal over the entire abdomen of the mouse was analysed.

### Statistical analysis

Experiment results obtained were statistically evaluated by simple t-test. Differences were considered significant if P < 0.05. Statistical analysis was performed by SigmaPlot 10.0.

## Abbreviations

s.c: Subcutaneous
i.p: Intraperitoneal

## References

[1] Pal T, Permuth-Wey J, Sellers TA (2008). A review of the clinical relevance of mismatch-repair deficiency in ovarian cancer. Cancer.

[2] Alberts DS, Liu PY, Hannigan EV, OToole R, Williams SD, Young JA (1996). Phase III study of intraperitoneal cisplatin-intravenous cyclophosphamide versus intravenous cisplatin-intravenous cyclophosphamide in patients with optimal disease stage III ovarian cancer: A SWOG-GOG-ECOG Intergroup study. Int J Gynecol Cancer.

[3] Ledermann JA, Kristeleit RS (2010). Optimal treatment for relapsing ovarian cancer. Ann Oncol.

[4] Bookman MA (2010). The addition of new drugs to standard therapy in the first-line treatment of ovarian cancer. Ann Oncol.

[5] Parmar MKB, Ledermann JA, Colombo N, du Bois A, Delaloye JF, Kristensen GB (2003). Paclitaxel plus platinum-based chemotherapy versus conventional platinum-based chemotherapy in women with relapsed ovarian cancer: the ICON4/AGO-OVAR-2.2 trial. Lancet.

[6] Markman M, Walker JL (2006). Intraperitoneal chemotherapy of ovarian cancer: A review, with a focus on practical aspects of treatment. J Clin Oncol.

[7] Schorge JO, Modesitt SC, Coleman RL, Cohn DE, Kauff ND, Duska LR (2010). SGO white paper on ovarian cancer: etiology, screening and surveillance. Gynecol Oncol.

[8] Lim MC, Song YJ, Seo SS, Yoo CW, Kang S, Park SY (2010). Residual cancer stem cells after interval cytoreductive surgery following neoadjuvant chemotherapy could result in poor treatment outcomes for ovarian cancer. Onkologie.

[9] Scott KA, Holdsworth H, Balkwill FR, Dias S (2000). Exploiting changes in the tumour microenvironment with sequential cytokine and matrix metalloprotease inhibitor treatment in a murine breast cancer model. Brit J Cancer.

[10] Al-Niaimi A, Manuelli B, Safdar N, Chappell R, Seo S, Kushner D et al. Survival with surgical versus medical treatment of patients with bowel obstruction secondary to ovarian cancer recurrence: A 15-year experience. J Clin Oncol. 2009;27(15).

[11] Connolly DC, Hensley HH (2009). Xenograft and transgenic mouse models of epithelial ovarian cancer and Non invasive imaging modalities to monitor ovarian tumor growth in situ -applications in evaluating novel therapeutic agents. Curr Protoc Pharmacol.

[12] Vanderhyden BC, Shaw TJ, Ethier JF (2003). Animal models of ovarian cancer. Reprod Biol Endocrinol.

[13] Hensley H, Quinn BA, Wolf RL, Litwin SL, Mabuchi S, Williams SJ (2007). Magnetic resonance imaging for detection and determination of tumor volume in a genetically engineered mouse model of ovarian cancer. Cancer Biol Ther.

[14] Dinulescu DM, Ince TA, Quade BJ, Shafer SA, Crowley D, Jacks T (2005). Role of K-ras and Pten in the development of mouse models of endometriosis and endometrioid ovarian cancer. Nat Med.

[15] Ingersoll SB, Yue P, Ahmad S, Turkson J, Edwards JR, Holloway RW. Molecular characterization of highly tumorigenic cell lines used in a xenograph model to investigate cellular therapy for the treatment of refractory ovarian cancer. J Clin Oncol. 2009;27(15).

[16] Cheng KW, Lahad JP, Kuo WL, Lapuk A, Yamada K, Auersperg N (2004). The RAB25 small GTPase determines aggressiveness of ovarian and breast cancers. Nat Med.

[17] Reed E, Yuspa SH, Zwelling LA, Ozols RF, Poirier MC (1986). Quantitation of Cis-Diamminedichloroplatinum-Ii (Cisplatin)-DNA-intrastrand adducts in testicular and ovarian-cancer patients receiving cisplatin chemotherapy. J Clin Invest.

[18] Lagona J, Fettinger JC, Isaacs L (2005). Cucurbit[n]uril analogues: synthetic and mechanistic studies. J Org Chem.

[19] Kim J, Jung IS, Kim SY, Lee E, Kang JK, Sakamoto S (2000). New cucurbituril homologues: syntheses, isolation, characterization, and X-ray crystal structures of cucurbit[n]uril (*n* = 5, 7, and 8). J Am Chem Soc.

[20] Kim J, Ahn Y, Park KM, Kim Y, Ko YH, Oh DH (2007). Carbohydrate wheels: cucurbituril-based carbohydrate clusters. Angew Chem Int Ed Engl.

[21] Plumb JA, Venugopal B, Oun R, Gomez-Roman N, Kawazoe Y, Venkataramanan NS (2012). Cucurbit[7]uril encapsulated cisplatin overcomes cisplatin resistance via a pharmacokinetic effect. Metallomics.

[22] Morton CL, Houghton PJ (2007). Establishment of human tumor xenografts in immunodeficient mice. Nat Protoc.

[23] Jensen MM, Jorgensen JT, Binderup T, Kjaer A (2008). Tumor volume in subcutaneous mouse xenografts measured by microCT is more accurate and reproducible than determined by 18 F-FDG-microPET or external caliper. BMC Med Imaging.

[24] Black PC, Shetty A, Brown GA, Esparza-Coss E, Metwalli AR, Agarwal PK (2010). Validating bladder cancer xenograft bioluminescence with magnetic resonance imaging: the significance of hypoxia and necrosis. BJU Int.

[25] Plumb JA, Milroy R, Kaye SB (1987). Optimization of a chemosensitivity assay based on reduction of the tetrazolium Dye, Mtt. Anticancer Res.

